# The First Steps to Building Research Collaborative Using Strength-Based Assessments and GIS Maps with a Sample of Community-Based Organizations in the Bronx, NY

**DOI:** 10.1089/heq.2023.0015

**Published:** 2024-01-23

**Authors:** María Isabel Roldós, Jaye Jones, Jocelyn Rajaballey

**Affiliations:** ^1^ City University of New York, Lehman College, Bronx, New York, USA.; ^2^ School of Health Sciences, Human Services and Nursing, Department of Health Equity, Administration and Technology, Lehman College, City University of New York, Bronx, New York, USA.; ^3^ City University of New York (CUNY) Institute for Health Equity, Lehman College, City University of New York, Bronx, New York, USA.; ^4^ Division of Students Affairs, Lehman College, City University of New York, Bronx, New York, USA.; ^5^ School of Natural and Social Sciences, Department of Earth, Environment and Geospatial Sciences, Lehman College, City University of New York, Bronx, New York, USA.

**Keywords:** Bronx, NY, collaborative building, health disparities, CBPR approach, strength-based assessment

## Abstract

**Introduction:**

Community-based participatory research (CBPR) is one of the most effective strategies for conceptualizing, developing, and executing programs or interventions that address health disparities in community settings. The City University of New York (CUNY)'s Institute for Health Equity (CIHE) focuses on the social determinants that affect the physical and mental health of New York City's poor and underserved.

**Methods:**

This study utilized a modified Strengths, Weaknesses, Opportunities, and Threats (SWOT) tool as a strength-based assessment (SBA) to evaluate community-based organization (CBO)'s *Areas for Growth* (SWOT-SBA). This approach was used to identify CBOs' strengths, prospects, and priorities to address the Bronx's health disparities. Furthermore, this study collected descriptive information on CBO's catchment areas, services provided, and population served to create interactive and static maps and contingency tables using the Arch-GIS software.

**Results:**

This study was the first step to building CIHE *Healthy-Bronx Research Collaborative* to address the Bronx's health disparities. The results indicate that Hunts Point and Longwood Community Districts are the most served by CBOs. The SWOT-SBA suggests that CBOs' engagement through “appreciative inquiry” to conduct a CBPR has the most promise for a successful partnership between CBOs, research partners, and local stakeholders.

**Conclusion:**

This analysis suggests that CBOs center their resources to function as a leader in the Bronx and have identified the need to expand services during the pandemic. Findings from this study suggest that CBOs want to collaborate in CBPR initiatives.

## Introduction

The Bronx is one of the five boroughs of New York City (NYC) and is considered the most densely populated county in New York State (NYS) and in NYC.^1^ According to the U.S. Census Bureau 2023 estimates, the population breakdown by race and ethnicity is the following: 8.7% of the borough's population self-identify as White non-Hispanic; close to 44% of the population is Black or African American non-Hispanic, 4% identify as Asian non-Hispanic, 3% are Native American, and more than 3% identify as mixed race. Furthermore, close to 60% of the population is of Hispanic or Latino origin, and more than 58% of the population report speaking a language other than English at home.^1^

The County Health Ranking and Roadmaps Ranking (CHR&R) has placed the Bronx as the least healthy county in NYS since 2010.^2^ It has the highest rates of asthma, diabetes, hypertension, obesity, tobacco use disorder, violent crime, and environmental contamination, compared with the other NYC boroughs and other NYS counties.^3^ COVID-19 compounded the health disparities experienced by Bronx residents.^4–6^ The neighborhoods hit the hardest were also those with the highest COVID-19 health-related outcomes, including case count, hospitalizations, and premature death.^7^ Of the top 15 most vulnerable neighborhoods in NYC, 9 are in the Bronx (Mott Haven/Melrose, Hunts Point/Longwood, Crotona/Morrisania, Concourse/Highbridge, South Fordham/University Heights, Belmont/East Tremont, Kingsbridge Heights/Bedford, Parkchester/Soundview, Baychester/Williamsbridge).^5^

During the pandemic, community-based organizations (CBOs) were called upon to join public health agencies, faith-based organizations, funders, health care services and agencies, and others to work collaboratively to face the challenges the pandemic brought.^6,8–10^ Some of the actions included increasing awareness of public health issues, increasing access to health services, reducing barriers to vaccines, among others.^11^ Public health agencies specifically targeted CBOs to assist in the promotion of public health directives. CDC's Community Guide for community partners provided informational resources and funding to engage in or support COVID-19 vaccination confidence activities and access in racial and ethnic minority communities.^12^

These efforts focused on racial and ethnic minority communities because of the disproportionate effect of COVID-19 on these groups and their experience of marginalization, discrimination, or disparities in receiving vaccines, or demonstrating hesitancy to get vaccines.^12^ To this end, CBOs with community-level reach were offered COVID-19 Vaccination Supplemental Funding, such as IP19-1901, CDC-RFA-1P21-2108, and CDC-RFA-DP-23-0014, among others.^13^ In the Bronx, for example, the CDC REACH project, titled Bronx Health Reach, activated their over 50 community partners to engage in COVID-related health initiatives.^14^ Bronx REACH's goal is to eliminate racial and ethnic disparities in health outcomes in diabetes and heart disease among African Americans and Blacks and Hispanic and Latinx communities in the southwest Bronx.^14^

The community-based participatory research (CBPR) approach centers on working with CBOs with community-level reach. Research suggests that CBOs are capable of serving at-risk community dwellers, addressing intervention challenges, and serving as liaisons between researchers, residents, and local authorities.^15,16^ CBPR is best achieved when academic researchers form collaborative partnerships with CBOs share the decision-making and co-lead the implementation with the goal of improving population health and promoting the widespread adoption of evidence-based programs and interventions.^17–21^

There is little literature on Bronx CBOs' capacities to engage in CBPR partnerships, and limited research has documented the effectiveness of their health promotion and disease prevention efforts using this approach. However, examples of meaningful collaborative efforts exist. For example, in the 1980s and 1990s, the Northwest Bronx Community and Clergy Coalition was created to address the crack cocaine epidemic among Bronx residents.^22^ For decades, grassroots community-based social mobilization efforts to address food insecurity, such as United Bronx Parents, Park Slope Food Coop, God's Love We Deliver, Community Food Resource Center, among others, have also proliferated.^23,24^

In 2022, the NYC Department of Health and Mental Hygiene conducted a COVID-19 and flu vaccine community project with barbershops, hair salons, beauty salons, nail salons, and faith-based organizations to identify potential partners in selected neighborhoods in the Northeast Bronx as “health champions” to increase COVID-19 and flu vaccine knowledge. Coalitions have also been formed to support immigrants moving into the Bronx and to create culturally sensitive educational programs on infectious and chronic diseases so faith-based organizations can promote prevention.^25^

Lehman College is the only senior college of the City University of New York (CUNY) system located in the Bronx and is considered an anchor institution in the borough.^26–29^ Lehman College established the CUNY Institute for Health Equity (CIHE) in 2009. CIHE's focus is the social determinants that affect the physical and mental health of NYC's poor and underserved communities.^30^ For over a decade, the CIHE has partnered with different Bronx coalitions to conduct community health projects. In 2020, the CIHE engaged in a visioning process and in 2021 re-established itself as a research institute with the mission to coordinate research, teaching, service, and community collaboration using data analytics to eliminate health inequalities and advance the science of health disparities.^31^

To this end, in 2021–2022, the CIHE launched an engagement process with Bronx CBOs to facilitate meaningful community connections, generate ideas, and engage with CBOs in joint initiatives that address Bronx health disparities. This study conducted a strength-based assessment (SBA) using a modified Strengths, Weaknesses, Opportunities, and Threats (SWOT) tool to focus on CBO's *Areas for Growth* (SWOT-SBA). This approach was used to depict CBOs' strengths, prospects for change, and to establish priorities to address the Bronx's health disparities. Furthermore, this study collected descriptive information on CBO's catchment areas, services provided, and populations served to create interactive and static maps and contingency tables using the Arch-GIS software.

## Materials and Methods

### Recruitment

This study conducted a SBA of a random convenient sample of CBOs in the Bronx. Upon approval from CUNY's Institutional Review Board (IRB), an online questionnaire was sent to CBOs across the Bronx to participate in CIHE's data call entitled the “Healthy-Bronx Research Collaborative.” CUNY's IRB awarded this project an expedited review under project number 2021-2056, and all CBO representatives signed an online consent form.

For CBO recruitment, the CIHE used lists of historical collaborations with CBOs and gathered lists of CBOs registered at various organizations and initiatives, including #not62 movement, NYC Department of Health and Mental Hygiene, the Bureau of Bronx Neighborhood Health initiatives, South Bronx Rising, Bronx Impact Collective, Bronx Health Reach, and other CBOs' coalitions databases. These organizations serve the community in the areas of housing, education, economic development, business development, health care, including mental health, counseling, and others. Two approaches were used to recruit CBOs into the study. The first approach was via e-mail, with an initial invite and weekly reminders for 4 weeks, and then every 2 weeks for 3 months.

Approximately 271 invitations were sent of which 48 responses were received. CIHE's research assistants followed-up with e-mails and calls to the CBOs to verify the organizational data. This effort resulted in 11 completed surveys. The second strategy was via a community organizer/convener based in the Bronx named, FBS. FBS was hired by the CIHE to collect the study's survey. To do so, FBS conducted door-to-door visits to CBOs and held town hall convening meetings. These meetings sought to create a meaningful conversation that generates ideas to address the Bronx health disparities. FBS engaged with more than 52 CBOs from CIHE lists and in the end 32 completed the CIHE survey.

### Measures and methods

Qualitative data were collected using an adapted SWOT analysis using a strength-based approach. SWOT-SBA adaptation framed questions as—Strengths: What organizations do well; Weaknesses (framed as *Areas for Growth*): What organizations need to grow their strengths; Opportunities: Where organizations can use their strengths to fill gaps; Threats: What are the obstacles to utilizing organizational strengths. Table 1 describes the SWOT-SBA instrument.

**Table 1.** SWOT-SBA and *Healthy-Bronx Research Collaborative Questionnaire*

SWOT-SBA questionnaire What programs, activities, or services does the organization do best? What do the organization's beneficiaries and clients say the organization does best? What advantages does the organization have compared with other similar CBOs? What obstacles does the organization face in the provision of programs, activities, or services? What knowledge, talent, and skills does the organization currently lack that would allow the organization to grow? In terms of the programs, activities, and/or services, how does the organization face challenge? What do the organization's beneficiaries say the organization needs to do better? What could the organization do today that is not already being done in terms of program activities or services? How is the field of service changing? How can the organization take advantage of these changes? What changes in the organization's field (e.g., in standards, policies, legislation, technology) potentially threaten future growth and/or success? Please list the organization's priorities in terms of health concerns and other issues prevalent in the Bronx. What does the organization envision the Bronx will look like in terms of health in 5 years?

CBO, community-based organization; SBA, strength-based assessment; SWOT, Strengths, Weaknesses, Opportunities, and Threats.

Table 2 presents the questions used to collect CBO's information. This includes general information about the organization; the organization's mission, programs, and services; population focus; and information on their catchment area and the identification of the community board district (CBD) of focus. Survey Monkey powered the online questionnaire.

**Table 2.** Healthy-Bronx Research Collaborative Survey Questions

Organization Name
Address, City, State
E-mail Address
Phone Number
Website Address
Does the organization have multiple locations?
Website listing Multiple Locations
Organization Contact Person
Contact Person Title
What population(s) does the organization serve? (Check all that apply) (adults, children, adolescents, young adults, women, veterans, disabled, elderly, homeless, other)
Does the organization focus on groups or populations that experience health disparities or health inequalities? (Yes/No)
If yes, which of the following health disparity populations does the organization serve? (Lower socioeconomic status, sexual and gender minorities, racial and ethnic minorities, underserved rural populations)
Please identify the organization's catchment area by community boards district(s) in the Bronx See NYC Planning Community Profiles website for reference
Do the services the organization provides fall under any of the following categories? (Check all that apply or list more under other) (primary care services, counseling services, food banks, employment counseling/placement services, housing stability services, education/training, other)
Please provide some details on the areas the organization is interested in collaborating in (research, advocacy)
Are you interested in hosting college student or interns? (Yes/No)
Is the organization seeking academic partners? (Yes/No)
Did COVID-19 have any of the following impacts on the organization's operations? (Decrease funding, increase funding, expansion of services, restriction of services, shift to remote services)

NYC, New York City.

### Analysis

The methodology used to process the SWOT-SBA responses was a thematic analysis approach.^32,33^ This process included the identification of recurrent words and phrases and the assignment of labels to those that represent a recurring theme in each SWOT-SBA category.^32^ These labels were later bundled into short phrases. Four independent coders analyzed SWOT-SBA responses, which include the authors and two Lehman College students. Descriptive information provided by CBOs was organized in contingency tables to depict CBOs' descriptive and organizational information using Excel and Arch-GIS software to locate CBOs in the Bronx by catchment areas, services provided, and population served.

## Results

CIHE sent more than 200 invitations to complete the online questionnaire and through a community-convener from the Bronx. A total of 44 CBOs completed the CIHE's contact information section, and 36 completed the contact information plus the services rendered section of the survey to build the GIS maps, and 34 completed the SWOT-SBA sections. Table 3 depict the descriptive information of the CBOs that completed the survey's contact information section of the survey. The results from this study are divided into four sections. Section 1 will illustrate the CBOs' location and catchment area in relation to the Bronx's CBD(s) using GIS maps; Section 2 will present the results of the SWOT-SBAs; Section 3 will describe CBO's health equity perspectives and views; and Section 4 will present CBOs' responses in relation to the COVID-19 pandemic.

**Table 3.** Descriptive Information of the Community-Based Organizations (*n*=44)

“Healthy-Bronx Research Collaborative”		
Years established (*n*=40)		
Before 1980	9	23%
1981–1999	11	27%
2000–2010	12	30%
2010–2020	8	20%
Years working in the Bronx (*n*=40)		
More than 5 years	36	90%
3–5 Years	2	5%
1–3 Years	2	5%
Population focus (*n*=42)^a^		
Adults	31	74%
Women	20	48%
Children	28	67%
Adolescents	31	74%
Young adults	35	83%
Veterans	9	21%
Elderly	20	48%
Homeless	12	29%
LGBTQ+	11	28%
Health disparity populations (*n*=33)^a^		
Racial and ethnic minorities	24	73%
Sexual and gender minorities	12	36%
Underserved/low socioeconomic status	29	88%
Rural	4	12%
Type of services provided (*n*=36)		
Primary care health services	3	8%
Counseling services	11	31%
Foodbank	9	25%
Employment/placement services	6	17%
Housing instability services	5	14%
Education/training	28	78%

^a^ CBOs were able to select multiple options. Categories were not mutually exclusive.

LGBTQ+, lesbian, gay, bisexual, transgender, queer, intersex, asexual, and others.

### Section 1: description of CBOs

**FIG. 1.**
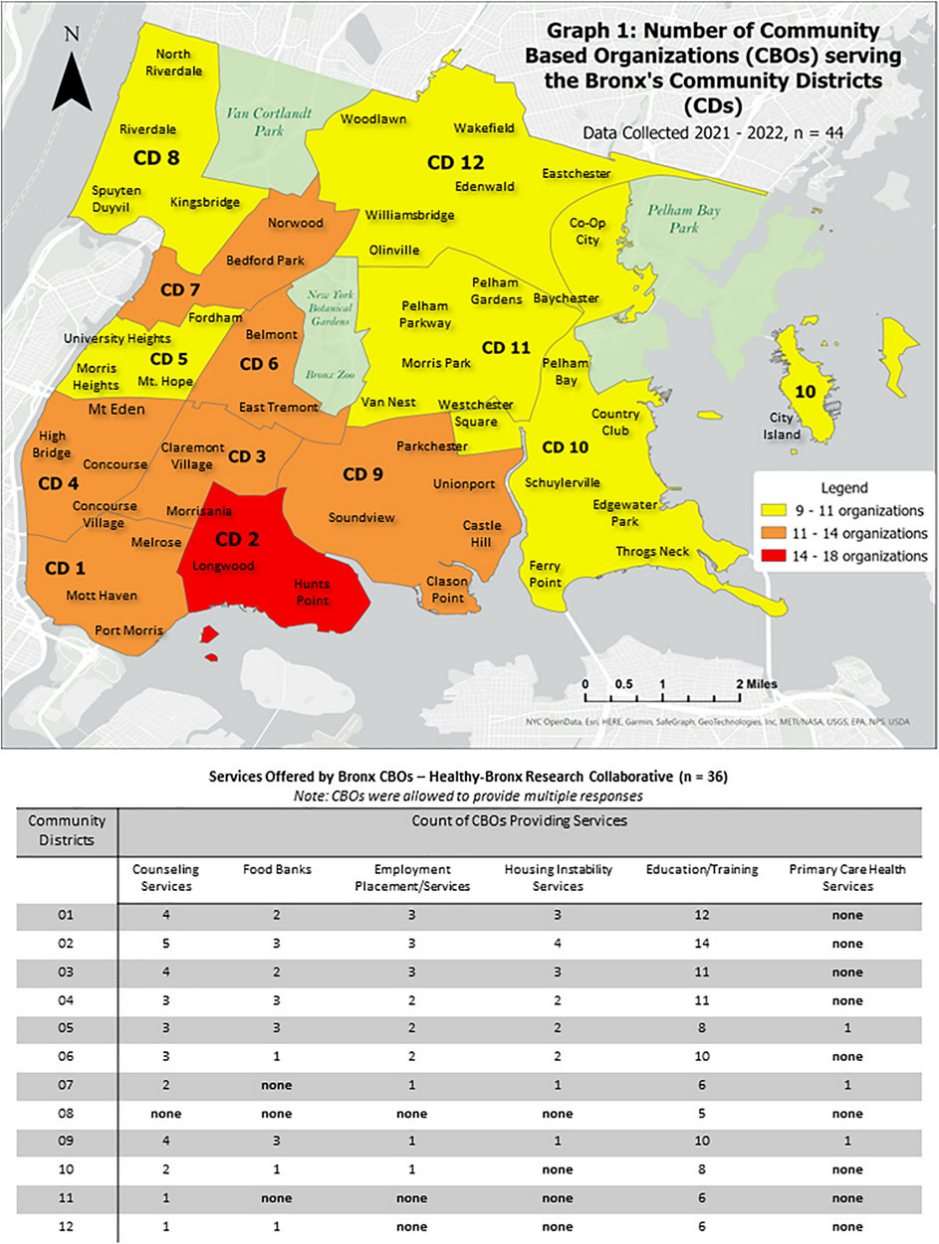
Number of CBOs serving Bronx by CB. CB, community board; CBO, community-based organization.

Descriptive results suggest that nearly 80% have been established in the Bronx for more than 10 years, and 5% report establishment in the last 3 years. CBOs report working with multiple populations and groups throughout the Borough. Results from the study also suggest that CBOs work in multiple CBDs and spread services and programs across multiple neighborhoods. Figure 1 presents the CBOs' location and catchment area for the services and programs provided. Of the 44 CBOs that responded the contact information and general description sections of the survey, 36 completed the sections of the services rendered.

Results indicate that education and training are the services most provided by CBOs, followed by counseling services, food banks or pantries, and housing support services. District 2 Hunts Point and Longwood is the community district most served, followed by District 3 that includes Claremont, Crotona Park East, and Morrisania, and District 4 includes Concourse and Highbridge. Furthermore, Figure 1 shows the type of services being provided by CBD. For example, for CBD 2, 18 CBOs reported working in these neighborhoods. In contrast, CBD 12, one of the biggest in the Bronx had nine CBOs reporting working on this locality and fewer services provided compared with other CBDs.

Findings also suggest that veterans, the elderly, and the homeless had the lower proportion of services provided. Sexual and gender minorities+, and rural populations and groups were the least mentioned. Moreover, most CBOs reported working with racial and ethnic minorities (73%) and underserved low-income individuals and families (88%). Furthermore, nearly half of the CBOs that completed the questionnaire (34 CBOs) indicated their intent to collaborate to conduct research (14 CBOs) and 16 CBOs to engage in advocacy initiatives. Close to 70% of the CBOs are seeking academic partners and/or willing to participate in a community advisory board, respectively.

### Section 2: SBA

The key trends identified in the SWOT-SBA are displayed in Table 4. The SWOT-SBA analysis provided organizations with an opportunity to identify their service provision, the allocation of resources, and challenges—both external and internal—and their ability to enact the organization's mission or vision.

**Table 4.** Key SWOT-SBA Themes Identified—SWOT-SBA—*Healthy-Bronx Research Collaborative* (*n*=34 Community-Based Organizations)

Strengths	Growth
Community engagement	Service expansion
Youth programming	Physical space
Housing	Fundraising
Food	Staffing
Education/training	Development

Opportunities	Obstacles for growth
CBO collaboration	Lack of funding
Increase of programming	Lack of physical space
Capacity increase	COVID-19
Competition	Policies
Employment trends	Organizational size

#### Strengths: what organizations do well

Findings from the qualitative SWOT-SBA analysis suggest that organizations provided a range of services that they felt were reflective of their strengths including those that addressed basic needs (e.g., housing and food insecurity), focused on community engagement (i.e., advocacy) and on programming that supported the educational, socioemotional, or health-related needs of adults and youth. This assistance was generally offered in a context tied to an organizational and personal history in the Bronx and included an understanding of the borough's culturally diverse neighborhoods. For example, one organization noted:

We are Bronx based and have deep roots in the communities we serve. Much of our staff comes from the community and has a strong connection to the families we serve. We have a good retention rate for our staff so many people in the community feel comfortable seeking help from people they know. We often serve multiple generations from a family with the different services we provide.

One CBO described themselves as “Locally supported, volunteer-driven, intergenerational, and consistent in services provided,” while another noted that:

The majority of our staff is multi-lingual, multi-ethnic, and representative of the community we serve. This is from line workers all the way up to the CEO, CMO …

This appreciation for history and place meant that organizations were prepared to provide the multidimensional supports community members required. Said one organization:

We provide wrap-around services and focus on families and individuals in a more holistic way than other organizations. Given how large we are and the multitude of services we provide, we can assist families and individuals with many of the challenges they are facing. We do not necessarily need to refer people to services outside of the organization.

This knowledge of the community was embedded in the social relationships that developed between organizations and their constituents and strengthened the sense of trust:

Our community says that our staff is both knowledgeable and kind. For them [name redacted] isn't just somewhere you get services, but a place where you build community and feel like you are family.

#### Areas for growth (weaknesses): what organizations need to grow their strengths

As organizations need to heighten their strengths, they centered on the need for additional financial supports and physical space. They also identified needing the means to build their fundraising skills and improve their ability to communicate and market their service to the broader community. This need for capacity-building illustrates some of the challenges to address these communities' multifaceted and systemic needs. Simultaneously, these complexities highlight the benefits of collective action as many organizations collaborate to extend their impact. For example, one small CBO succinctly noted the need for:

Space, adequate human personnel and funding.

A health-based CBO tied their growth to additional funding:

[Our] beneficiaries wish we could expand those “out of the box” services that help support the vulnerable members of the community. They would like us to increase capacity. In order to do so we need increase grant funding.

Another organization focusing on making their services more visible stated:

We need to learn to promote ourselves and communicate with donors. It is something that has fallen by the wayside given the small team and only one person really fundraising.

Despite these concerns, organizations felt able to draw on their assets—internal and community-based—to address these challenges. One religious organization declared:

We look inward to draw strength in the face of challenges and seek collaboration with other providers/community partners.

Another described how:

As a team we come together to brainstorm the challenges and try to create and establish and resolution. Depending on the challenge we will also reach out to the community to ask for their feedback and support.

#### Opportunities: where organizations can use their strengths to fill gaps

Organizations identified the expansion of services, encouraging collaboration and capacity-building and providing more virtual/online services as opportunities for utilizing their strengths to better serve the community, especially those facing the most challenges. One organization asserted:

We need to expand our support services. Those intended for the most vulnerable patients … [And] provide additional assistance such as developing community housing, case management services, and counseling in one location.

While another documented the expansive possibilities of the digital realm:

The use of virtual spaces has expanded digital access. We started as a Bronx-specific org but now we welcome people from across the country and internationally.

Linked to these concerns was the intersection of basic needs, health equity, and racial and economic justice, which the disproportionate impact of the pandemic on the Bronx has heightened. As a result, some organizations emphasize how their strengths align with the social determinants of health to better tackle complex community needs. One CBO that provided a range of services including afterschool programs and mental health counseling, acknowledged:

Our current strategic plan is pushing [the organization] to be more focused on the interconnectedness of domestic violence, housing instability and job stability.

A different program highlighting the importance of collaboration maintained that:

The needs for families continue to exist and the pandemic has introduced additional challenges to families. As an organization in the service field, [we] feel that we need to continually evolve to meet those needs, as best we can or connect with other CBOs that provide that in the community.

The trend toward partnerships was something that several organizations felt would have a positive impact on communities, said one:

“We are noticing organizations are now coming closer together and wanting to partner for funding opportunities to make RFPs stronger and create a collective. This is something we are very eager to be on board with!” Another described their interest in partnering with “community gardens” and other CBOs around “various advocacy issues” to host community events and provide constituents with more comprehensive support.

#### Threats: what are the obstacles to utilizing organizational strengths

The challenges organizations faced were limited physical space and funding; staff shortages and access to technology were highlighted as obstacles to service provision, expansion, and collaboration. One organization listed the following barriers:

Lack of space to provide services, concerns about funding, attracting new staff from the community.

The fact that smaller organizations that were more embedded in the community were unable to compete for the funding that could help them expand was noted by one CBO:

Larger organizations that have no long-standing relationship with local communities are the ones receiving funding for services in communities of color and the bulk of such funding goes into personnel and operational expenses and is not invested in the local community and its people.

In addition, limited access to digital platforms was an issue, one organization acknowledged:

Technological limitations can potentially threaten the future growth and/or success of the organization. Social media platforms are changing exponentially and it's harder to catch up with said changes.

### Section 3: health equity findings

CBOs ranked crime, chronic diseases and illness (diabetes and asthma), and mental health as the top 3 health outcomes of most significant concern related to health equity. Responses include:

The Bronx priorities are in food insecurity; Food insecurity, depression, financial anxiety, hopelessness, diabetes and high blood pressures are among the issues the population we serve suffer from.

Regarding envisioning what health equity looked like for the Bronx, CBOs in the study favored interventions and programs that reduced crime and promoted greener spaces. One organization noted:

We firmly believe that there will be a community free of violent crime that allows for a harmonious relationship between and among residents in the Bronx County; less crime in the community and better partnership with NYPD and CBOs to help facilitate conversations.

Study focused on the strengths and areas of growth of CBOs. CBOs' vision of the future lie in environment justice, social engagement, and health equity:

more green spaces and equitable funding from the city to host health and wellness programming; reduce stigma around mental health, more green spaces and community building happening within neighborhoods and the borough as a whole.

### Section 4: CBOs' response to COVID-19

CBOs' response to COVID-19 was assessed in close-ended question as well as part of the SWOT-SBA. CBOs had the following options to describe their response and impact of the COVID-19 on their organization: decrease funding, increase funding, expansion of services, restriction of services, shift to remote services. All CBOs responded experiencing all the options of the COVID-19 question. Responses suggest that CBOs received new sources of income to increase COVID-19 awareness and increase vaccination and to promote health, as well as reporting shift of their services from those detailed in their mission to COVID-19-related activities. CBOs also reported loosing funding and reducing services. Responses indicate that CBOs faced many challenges and adapted their activities to meet the urgent needs of populations. One organization noted the following:

The Covid-19 pandemic exacerbated some of the health disparities our communities chronically suffer from, and it prompt[ed] many to finally pay attention. We believe this is a change that could potentially bring more funding for services in our community.

## Discussion

Overall, findings from this study suggest that CBOs in the Bronx have strong capabilities in promoting and communicating their strengths and vision of the Bronx. The SWOT-SBA analysis suggest that organizations provided a range of services that they felt were reflective of their strengths including those that addressed basic needs in the context of the CBOs' organizational and personal history in the Bronx, which include their understanding of the borough's culturally diverse populations and groups, and their relation with public authorities. CBOs' reflections centered on their resources to function as a leader in the Bronx, which include financial supports and physical space. Finally, organizations identified the expansion of services, encouraging collaboration and capacity-building and providing more virtual/online services as opportunities for utilizing their strengths to better serve the community.

The Hunts Point and Longwood Community Districts (CD2) are the most served by CBOs. These neighborhoods are low-income, residential areas largely made up of Hispanic or Latinx communities. Data from the latest NYC Community Profiles 2018 suggest that Hispanics or Latinx groups represent 74% of the population in these neighborhoods and are primarily families of Puerto Rico descent or origin, followed by Dominicans and Mexicans. African Americans or Blacks are the second largest racial minority with 21% of the population,^34^ Compared with the Bronx as a whole, and NYC, Hunts Point and Longwood neighborhoods have the highest poverty rate (29% compared with 25% in the Bronx, and 20% in NYC); the highest rent burden with 58% in Hunts Point compared with 51% in NYC. CD2 was also one of the neighborhoods with the highest number of COVID-19 infections and deaths. This is consistent with CBOs' report of receiving more resources and income during this time, as those resources may have been directed to COVID-19-related activities and initiatives, such as food banks, vaccinations awareness, and mental health services.

CBOs' testimonies suggest that they are likely to engage in CBPR and projects, as most CBOs strongly intend to collaborate with academic partners and experts in community health. CBOs can envision a Bronx without health inequities and perceive violent crime prevention and access to green spaces as the areas where Bronx CBOs should focus. The CBOs from this study provided the most services in the following areas: education and training; counseling services; housing instability services. Findings from this study suggest that the services and programs provided by the CBOs are aligned with the population's needs and sociodemographic composition.

The use of SWOT-SBA provided a forum for CBOs to engage in “appreciative inquiry”^35^ and allowing them to draw on their areas of strength, success, and promise as a way to highlight the possibilities for community-based change. In many ways, the Bronx has been defined by its challenges and deficits (e.g., the poorest county in the state) with conversations about health equity emphasizing the residents' lack of access or the need to “close gaps.” The SWOT-SBA started where the community was and encouraged them to illuminate their capacities, available resources, and desire to create partnerships.^35,36^ This focus can strengthen CBPR by expanding what equity-centered health and wellness solutions look like.^35,36^ Indeed, this study confirmed the importance of CBO alliances and innovation when public health emergencies, such as COVID-19, arise. CBOs reported being resilient and empowered in their mission and COVID-19 served as a platform for them to leverage a range of assets—collaborative, internal and external—to address the multiple consequences of the pandemic, including mental health, food, and economic insecurity.

## Conclusion

The Bronx has a long history of social advocacy and the creation of coalitions to address the many challenges Bronx residents have endured overtime. Addressing the health disparities in the Bronx will require an open discussion of unequal policies related to housing, education, the environment, and the criminal justice system that have perpetuated disparities. The direction of the Bronx can only be built with a meaningful analysis of the health burden or epidemiological transition of the Bronx and in the identification of the strengths of CBOs with community-reach.

Academic and research organization are best positioned to advise coalitions on assessments, evaluation, and best practices for project implementation. CBOs in the study indicate their intent to collaborate between them and with academic partners. Results from the SWOT-SBA and CBOs response to COVID-19 crisis suggest that CBOs are inclined to a CBPR approach to creating a coalition to address the Bronx health disparities. The long-term goal of this project is an alliance of CBOs that will join CIHE's Community Advisory Board and conduct CBPR initiatives to address the borough's health disparities.

Future work includes further strengthening CBOs by identifying areas for growth, including business management, GIS mapping, and data analytics.

## Abbreviations Used

CB: community board
CBD: community board district
CBO: community-based organization
CBPR: community-based participatory research
CIHE: City University of New York Institute for Health Equity
CUNY: City University of New York
IRB: institutional review board
NYC: New York City
NYS: New York State
SBA: strength-based assessment
SWOT: Strengths, Weaknesses, Opportunities, and Threats
